# The Mechanisms Underlying Interference and Inhibition: A Review of Current Behavioral and Neuroimaging Research

**DOI:** 10.3390/brainsci11091246

**Published:** 2021-09-20

**Authors:** Oliver Kliegl, Karl-Heinz T. Bäuml

**Affiliations:** Department of Experimental Psychology, Regensburg University, 93040 Regensburg, Germany; karl-heinz.baeuml@ur.de

**Keywords:** human memory, interference, inhibition

## Abstract

The memory literature has identified interference and inhibition as two major sources of forgetting. While interference is generally considered to be a passive cause of forgetting arising from exposure to additional information that impedes subsequent recall of target information, inhibition concerns a more active and goal-directed cause of forgetting that can be achieved intentionally. Over the past 25 years, our knowledge of the neural mechanisms underlying both interference-induced and inhibition-induced forgetting has expanded substantially. The present paper gives a critical overview of this research, pointing out empirical gaps in the current work and providing suggestions for future studies.

## 1. Introduction

Since its inception in the late 19th century, memory research has attempted to identify the factors that lead to forgetting. Until the 1980s, the prevailing belief was that forgetting is primarily generated by passive or incidental environmental factors. One such factor is the length of the retention interval between study and test, with research showing that memory of previously studied target information often declines as the retention interval between study and test is increased [1]. Another prominent factor is interference, with studies showing that recall of previously studied information can suffer on a retention test when additional nontarget information has been encoded prior to the test [2,3]. Interference effects in particular have been investigated extensively during the first two-thirds of the 20th century.

The two best-researched types of interference-dependent forgetting are retroactive and proactive interference. The first demonstration of retroactive interference stems from Müller and Pilzecker [2], who showed that memory of some originally studied target information was worse when the study of that information was followed by the study of interpolated nontarget information. The Müller and Pilzecker finding provided the initial impetus for an abundant number of studies on the role of retroactive interference for forgetting. Retroactive interference was basically considered the major cause of memory failure [4,5], when in 1957, Underwood [6] published a paper in which he cast doubt on the notion that forgetting is primarily the result of detrimental effects of subsequent learning of nontarget information. Instead, he made the argument that, in many cases, forgetting can be caused by the detrimental effects of nontarget information studied prior to the study of the target information, i.e., proactive interference. Since this seminal publication, proactive interference has been broadly studied, with the results confirming Underwood’s notion that proactive interference can arise over a wide range of materials and settings (for reviews, see [7,8]).

The two most prominent experimental tasks to examine both retroactive and proactive interference are paired-associate learning and multiple-list learning. In paired-associate learning, participants may initially study a first list of stimulus-response word pairs (e.g., *house*–rent, or A-B) and then a second list of pairs where the same stimulus word is presented as in the first list but a new response word is coupled with each stimulus word (e.g., *house*–lease, or A-D). On the final test, the `A’ stimulus word is shown as a cue (e.g., *house*–?) and, in the retroactive-interference version of the task, participants are asked to recall the response word of the first list (rent, or the `B’ response) whereas, in the proactive-interference version, they are asked to recall the response word of the second list (lease, or the `D’ response). Recall of both the `B’ and the `D’ responses is typically impaired when compared to a control condition in which both the stimulus and response words of the second list pairs are changed (e.g., *earth*–round; *table*–cook, or A-B, C-D), thus reflecting retroactive and proactive interference [9,10].

In contrast, in multiple-list learning designs, participants may study a target list of unrelated items (e.g., *nose*, *wind*, *mouse*, etc.) and are then tested on it. Retroactive interference in this task is reflected in the finding that recall of the target list is usually worse when, following the study of the target list, additional nontarget lists are studied, compared to when subjects engage in unrelated distractor activities for the same duration of time. Proactive interference in this task is reflected in the finding that recall of the target list is typically worse when, prior to the study of the target list, additional nontarget lists are studied, compared to when subjects engage in unrelated distractor activities for the same duration of time [11].

Even though paired-associate learning and multiple-list learning differ in critical aspects, there is overlap in the types of processes that are thought to cause retroactive and proactive interference in the two tasks. In particular, retrieval explanations assume that, in both tasks, additional study of nontarget information—be it before (retroactive interference) or after (proactive interference) the study of the target information—impedes participants’ ability to access the target information at the time of test, thus causing a less effective memory search and reducing the probability that the target information will be produced [3,8,12,13,14]. This explanation, similar to most other accounts of interference-induced forgetting, suggests a passive or incidental cause of forgetting: Forgetting arises because environmental circumstances have changed.

However, the view that more active factors can also contribute to forgetting and thus enable our everyday functioning was already implied 80 years ago by famous Argentine author Jorge Luis Borges. In his short story *Funes, the memorious* (1942), Borges [15] depicts Funes, a character who can record any impressions he encounters and reproduce them in the smallest detail but who, at the same time, is incapable of remembering, thinking, or acting properly. Borges illustrates that an absolute memory without selection or forgetting leads to a form of paralysis—or blindness—of the mind. Studies of individuals with Savant syndrome who exhibit similar characteristics to those of Funes, certainly support Borges’ assessment that without forgetting, we would no longer be able to function adequately in everyday life [16]. While the view that forgetting can also occur as a result of active suppression or inhibition mechanims had been expressed in some cases before the 1980s [10,17], it was not until the influential work of Robert Bjork and his colleagues [18,19,20] on goal-directed forgetting that the inhibition idea gained traction.

Bjork et al. argued that there are situations in which forgetting serves an obvious purpose because the to-be-forgotten information is outdated. In everyday life, this may occur when, for instance, we read some information in the news that we later learn was incorrect or when a friend has relocated, and her old home address now has become irrelevant. In these types of situations, active and intentional forgetting of the outdated information has been shown to reduce (proactive) interference, thus allowing for a more targeted retrieval of the newer information. In the laboratory, such goal-directed forgetting has been examined with the list-method directed forgetting (LMDF) task. In this task, subjects typically study two lists of items and, after study of list 1, they are told to either forget list 1 or to keep remembering list 1 for a later test. List 2 is always to-be-remembered. A later test on both study lists typically shows that a cue to forget list 1 leads to impaired list-1 recall and improved list-2 recall, relative to a cue to keep remembering list 1. LMDF effects were originally explained with the selective-rehearsal account, which assumes that a cue to remember list 1 induces more mental rehearsal of list-1 items during list-2 encoding, whereas a cue to forget list 1 prevents such rehearsal, thus reducing later recall of list 1 [21]. Because the selective-rehearsal account cannot explain a couple of LMDF findings—such as the absence of LMDF in recognition tests [22]—Bjork et al. later argued that the list-1 forgetting, which is observed in this task, results from retrieval inhibition—a failure to access the list-1 retrieval route.

More recent research on so-called retrieval-induced forgetting (RIF) suggests the existence of interference situations that trigger an inhibitory mechanism, which affects the representation of an item in memory itself [23]. RIF is often examined with the retrieval-practice task, in which a subset of previously studied items from a semantic category is repeatedly retrieved (e.g., fruit–Or___), and the effect of this manipulation on later recall of the practiced and unpracticed material (e.g., fruit–Orange, fruit–Banana) is examined. Recall performance on a subsequent retention test on both practiced and unpracticed items usually indicates that memory of practiced items is improved relative to control items from a different semantic category (e.g., sports) and, critically, memory of unpracticed items is impaired relative to control items from a different semantic category, reflecting RIF. RIF is generally consistent with the blocking account [24], which builds on classic interference theory [12] and assumes that strengthening some material via retrieval practice causes interference of these items when an unpracticed item is later tested, thus impairing memory of the unpracticed items. As an alternative account, Anderson et al. (1994) [23] suggested that RIF may rather arise because, during initial retrieval practice, not-to-be retrieved material interferes and is actively inhibited to reduce the interference, thus resulting in impaired memory of the unpracticed items on the later retention test.

Because LMDF and RIF represent somewhat different experimental situations, there are commonalities and distinctions regarding the mechanisms that have been suggested to underlie the two forms of forgetting. In both tasks, the target of the forgetting is the irrelevant material—not the relevant material such as in interference tasks—and some form of inhibition is assumed to induce the forgetting. However, while in LMDF, inhibition of the irrelevant forget-cued list-1 items has been suggested to impair retrieval of the whole list-1 unit [20], inhibition of the irrelevant unpracticed items has been argued to affect the representation of those items directly by reducing their activation level in memory [25].

The goal of this review is to provide an overview of the last 25 years of research on the neural mechanisms underlying interference-induced and inhibition-induced forgetting. To this end, we first introduce the main tasks that have been applied to induce the two types of memory failure, i.e., paired-associate learning and multiple-list learning for interference-induced forgetting and the retrieval-practice task and the directed-forgetting task for inhibition-induced forgetting. For each task, we then provide an overview of critical findings and suggested cognitive mechanisms before we review the current knowledge on neural processes underlying the two types of forgetting. A final summary section will discuss the main insights on the two types of forgetting, emphasize empirical gaps in the current work, and offer suggestions for future studies.

## 2. Mechanisms Underlying Interference-Induced Forgetting

### 2.1. Paired-Associate Learning

**Figure 1:**
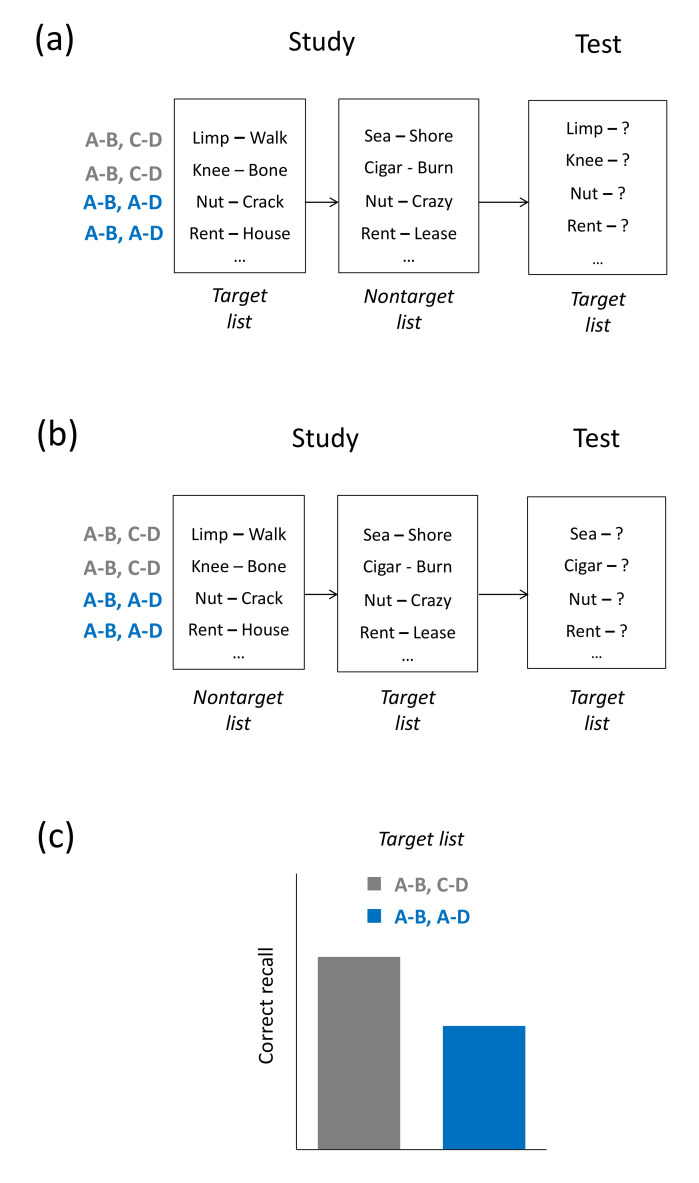
Paired-associate learning. (**a**,**b**) Retroactive-interference version (**a**) and proactive-interference version (**b**) of the task: Participants study a target list of stimulus–response pairs that is either followed (**a**) or preceded (**b**) by a list of other (nontarget) pairs. In both versions of the task, the second list consists of two types of word pairs: non-overlapping, new pairs for which neither the stimulus word nor the response word had appeared in the nontarget list (A-B, C-D); and overlapping pairs for which the stimulus word had already appeared in the nontarget list but the response word is new (A-B, A-D). (**c**) Typical results: In both the retroactive-interference and proactive-interference versions of the task, recall of the target list in A-B, A-D pairs is typically impaired relative to A-B, C-D pairs.

Paired-associate learning was first introduced in the late 19th century [26] and has since become one of the most applied learning tasks in memory research. In the retroactive-interference version of the task, participants initially study a first (target) list that may comprise stimulus-response word pairs (e.g., *house*–rent, or A-B) and then a second (nontarget) list consisting of additional pairs. The list-2 pairs are either overlapping or non-overlapping. In overlapping pairs, the first-list stimulus word is repeated and a new response word is associated with the stimulus word (e.g., *house*–lease, or A-D), whereas in non-overlapping pairs, both the stimulus and response words are new (e.g., *earth*–round; *table*–cook, or A-B, C-D). Some studies include a third type of list-2 pairs, in which the list-1 pairs are simply repeated (A-B, A-B). On a later test, the stimulus word is shown and the first-list response word (the `B’ response) needs to be recalled, with results typically showing that recall of the first-list responses is impaired for the overlapping (A-B, A-D) pairs when compared to the non-overlapping (A-B, C-D) pairs or the repeated (A-B, A-B) pairs. The study phase of the proactive-interference version of the task is identical to the retroactive-interference version, but at the later test, participants are asked to retrieve the second-list response word (the `D’ response). Analogous to the retroactive-interference version, recall of the second-list responses is often found to be impaired for overlapping pairs relative non-overlapping or repeated pairs, thus reflecting buildup of proactive interference [9,10] (see Figure 1a–c). Interference effects in paired-associate learning have been found to occur across a variety of study materials, including syllable–noun pairs [27], noun–noun pairs [28], odor–picture pairs [29], picture–word pairs [30], or pairs of colors and color words [31].

A classic account of interference effects in paired-associate learning is response-competition theory [12]. The account assumes that memory failure can occur at the test because the to-be-recalled target information is blocked by competing information. In overlapping pairs, two different responses, `B’ and `D’, are learned together with the same stimulus `A’, and when the stimulus `A’ is later at test presented as a retrieval cue for the `B’ target response in the retroactive-interference version, the unwanted `D’ response may be retrieved and block retrieval of the target `B’ response. Such competition would lead to worse memory of the `B’ response than in non-overlapping pairs, in which the `B’ response is only linked to a single stimulus. For the proactive-interference version, the response-competition account makes analogous assumptions. Evidence for the view that competition contributes to interference-induced forgetting comes from studies showing that recall of the `B’ response decreases as the number of A-D study cycles is increased (retroactive-interference version), and recall of the `D’ response decreases as the number of A-B study cycles is increased (proactive-interference version) [11,32,33,34,35] (but see [36,37]). Indeed, in the retroactive-interference version, repeated study practice of A-D pairs should increase the strength of the `D’ response relative to the `B’ response, thus enhance competition from the `D’ response and lead to the observed pronounced forgetting of the `B’ response. An analogous explanation applies to the effects of the repeated study of A-B pairs in the proactive-interference version of the task.

More recent work has argued that a critical factor modulating the degree to which retroactive and proactive interference arises in paired-associate learning is whether or not reminding of the first-list response occurs during the study of the second-list pairs and at the later test. In particular, it is assumed that when reminding occurs during the study of list 2—i.e., when a change in responses is detected—retrieval of the list-1 response may facilitate establishing an integrated memory representation that embeds the list-1 response together with the list-2 response, in conjunction with information about the order in which the two responses occurred [28,38]. Detection of change is assessed by asking subjects during encoding of list 2 to indicate pairs for which responses have changed (A-B, A-D) and to produce the list-1 (B) response. Successful recall of the B response is seen as an indication of change detection. Recollecting the recursive reminding during the later retention test can then prevent interference effects and instead even induce retroactive or proactive facilitation, i.e., improved recall for overlapping pairs, relative to non-overlapping pairs. Indeed, integrated memory traces containing both responses and their relative presentation order can provide additional cues for retrieval and, thus, facilitate recall of the target response. Previous behavioral work supports the reminding framework by demonstrating, for instance, that detection and recollection of change improves discrimination of the two study lists at test by increasing the probability that subjects correctly attribute a pair as having been shown as part of the target list [39].

### 2.2. Neural Correlates

Employing PET and fMRI, two early imaging studies investigated the neural processes underlying proactive interference in paired-associate learning. While PET has been applied during encoding, fMRI has been used during both encoding and during testing. In the first study, PET was applied while subjects engaged in a paired-associate learning task [40]. Results showed that activity in the ventrolateral and dorsolateral prefrontal cortex (PFC) was increased when participants encoded the A-D pairs during the study of list 2, relative to when they encoded completely new C-D pairs or when the A-B pairs were simply repeated. The researchers suggested that the lateral PFC may be engaged when associative semantic processing is necessary in creating a new (A-D) link between a stimulus and response word in the context of an already existing (A-B) link between the same stimulus and a different response.

Applying fMRI during encoding, Henson et al. (2002) [41] showed that, consistent with the Dolan et al. (1997) [40] findings, study of an A-D pair after prior study of an A-B pair increased activation in the inferior frontal cortex—including aspects of the lateral PFC—relative to an A-B control condition in which a stimulus word was presented only once with a response word. More important, Henson et al. also applied fMRI during the later cued-recall test. To prevent issues with image artifacts that typically arise during such memory tests, the stimulus words were presented as cues during a break between image acquisitions. Results showed that similar areas exhibited interference effects as during the study, with increased activity in the lateral PFC when subjects were asked to recall A-D pairs after prior A-B encoding. Because activity in the lateral PFC tends to increase with the number of responses that are associated with a common stimulus item [42], activity in this area may reflect greater difficulty in choosing a target response in the presence of competition from other responses.

Applying fMRI during encoding, a number of more recent studies have examined the processes underlying retroactive-interference effects in paired-associated learning. In a first study, Kuhl et al. (2010) [43] investigated how neural mechanisms active during the study of A-D nontarget pairs relate to later retention of originally studied A-B target pairs, using a variant of the A-B, A-D task in which study of the A-B pair was followed by either an A-D pair or no additional pair. At the behavioral level, results demonstrated that recall of the `B’ responses was worse in a later test when an A-D pair was present during the study than when it was absent, reflecting typical retroactive interference. At the neural level, results showed that greater activation in the posterior hippocampus and the parahippocampal cortex during the encoding of the A-D pairs predicted later retention of the `B’ responses. These results are consistent with computational theories of hippocampal function [44], which suggest that A-B pairs can be reactivated during A-D encoding, thus facilitating later memory of the A-B pairs. Alternatively, the results may point to pattern separation of the two lists resulting in distinct representations of list-1 and list-2 memories.

In a second study, Richter et al. (2016) [45] ran an fMRI experiment in which instructions guided subjects to either encode the A-D pair, recall the previous A-B pair, or integrate both pairs during learning of the A-D pair. Multivariate pattern classifiers were subsequently trained to discern the instructed processing states based on patterns of fMRI activity acquired during the study of A-D pairs. The results of this analysis showed that it was possible to distinguish the integration state from both the encoding and retrieval states. In particular, Richter et al. demonstrated that for a new sample of subjects who did not receive any instructions that would influence them to apply either an encoding, retrieval, or integration state, the decoding algorithm could successfully predict performance in an integration test—a test of the ability to correctly identify the `B’ response when probed with the `D’ response. Both the hippocampus and medial PFC were found to distinctly index subjects’ mnemonic processing states, with hippocampus activity patterns indexing the tradeoff between encoding and retrieval states and medial PFC being critically involved in reactivating older memories in the service of integration. [46] followed up on this research by applying once again the decoding algorithm from the sample of Richter et al. (2016) [45] to demonstrate that integration of both the `B’ and `D’ responses during A-D encoding—and not simply reactivation of the `B’ response during A-D encoding—is necessary to reduce interference.

### 2.3. Interim Summary

In paired-associate learning, behavioral work has found both retroactive and proactive interference to arise because, at the time of the test, responses from the nontarget list compete with the responses from the target list. The response-competition view is supported by behavioral work systematically manipulating the strength of the nontarget material. Behavioral work further indicates that retroactive and proactive interference effects can be tempered, or even reversed, when detection of change occurs during encoding of list-2 pairs—together with the recollection of this change at test. Regarding imaging work on proactive interference, the response-competition is in line with PET and fMRI studies showing that prior nontarget learning leads to increased activity in the lateral PFC, a brain region that has been suggested to be involved when a selection among competing information is required during demanding retrieval tasks. More recent work applying the retroactive-interference version of paired-associate learning has shown that the hippocampus and medial PFC may constitute neural substrates of reminding and memory integration. In addition, there is evidence from fMRI research using pattern classifiers that the detrimental effects of subsequent nontarget learning can be reduced when the target material is integrated with the nontarget material.

### 2.4. Multiple-List Learning

**Figure 2:**
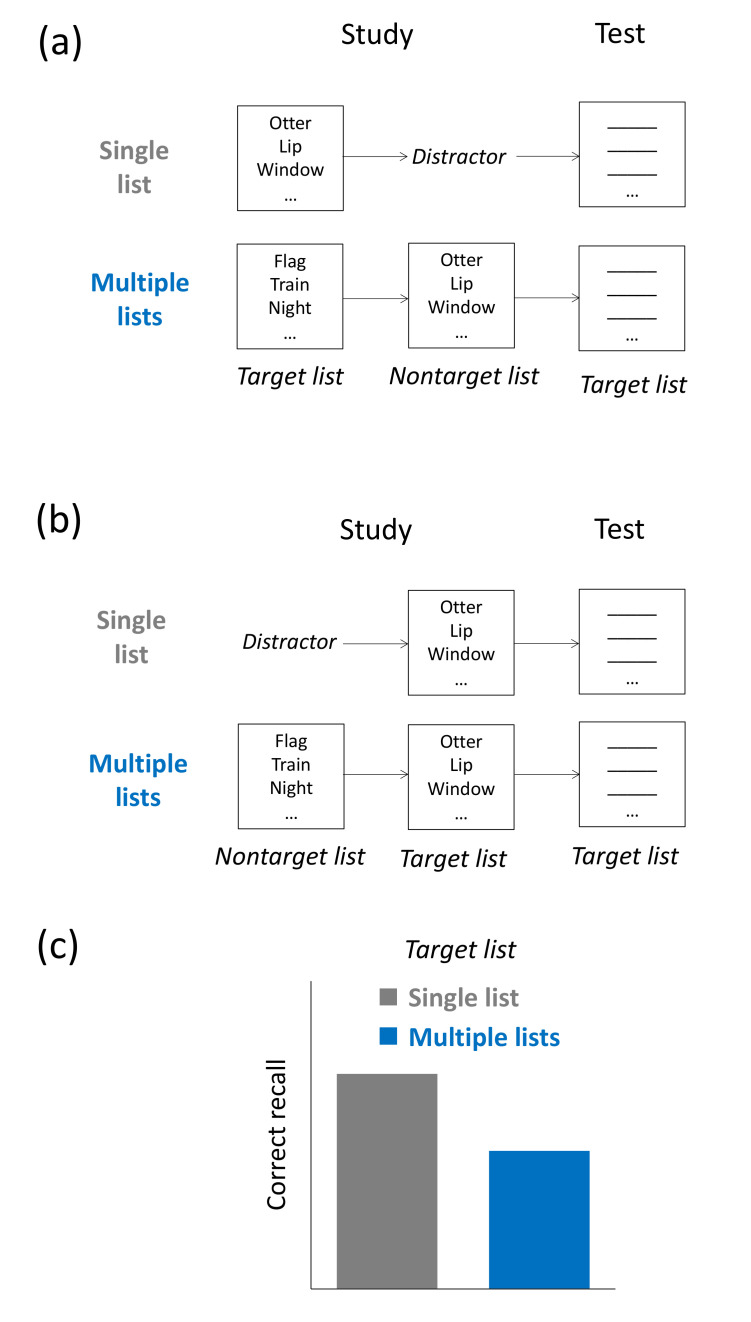
Multiple-list learning. (**a**,**b**) Retroactive-interference version (**a**) and proactive-interference version (**b**) of the task: Participants study a target list, which may consist of unrelated items. Study of the target list is either followed (**a**) or preceded (**b**) by unrelated distractor activity or the study of one or several additional (nontarget) lists. (**c)** Typical results: In both the retroactive-interference and proactive-interference versions of the task, target-list recall is typically impaired in the presence of additional nontarget encoding.

A second prominent task used to induce retroactive and proactive interference is multiple-list learning. Interference effects in this task are usually investigated by having participants study a target list, consisting, for instance, of unrelated nouns (e.g., *hand*, *storm*, *table*, etc.). In the retroactive-interference variant of the task, study of the target list is followed by the study of additional nontarget lists, whereas in the proactive-interference variant, study of the target list is preceded by the study of additional nontarget lists. These nontarget lists often consist of the same type of study material as the target list (e.g., unrelated nouns). In a later retention test, participants are asked to recall as many target items as possible. Retroactive and proactive interference in this task is reflected in the finding that the subsequent or prior study of nontarget material impairs recall of the target list in a later test, relative to a distractor condition in which study of the nontarget lists was replaced by unrelated distractor activities (e.g., simple arithmetic tasks) [27] (see Figure 2a–c). To our knowledge, retroactive and proactive interference in multiple-list learning thus far has mostly been demonstrated using words as study material.

Temporal discrimination theory has been suggested as a retrieval account of interference effects in multiple-list learning. The account assumes that the additional study of nontarget material impedes participants’ ability to constrain their memory search to the target-list items at the time of the test. As a consequence, they tend to include items from the nontarget lists into their mental search set, thereby impairing recall of the target items. In support of this idea, previous studies have shown that, for both the retroactive-interference and proactive-interference variants of the task, additional encoding of nontarget material not only decreases recall totals when the target list is tested but, critically, also increases mean response latencies [47,48,49]. Because response latencies—which measure the mean duration between the first item that is produced during the test and each subsequently produced item—have been shown to constitute a reliable measure of participants’ search set size at test, the findings suggests that additional nontarget learning leads to an enlarged mental search set size. Providing further support in favor of the temporal discrimination view, Unsworth et al. (2013) [49] were able to show that intrusions from nontarget material were increased during target-list recall in the presence of either retroactive or proactive interference.

Proactive interference in multiple-list designs has also been suggested to arise due to a failure at the encoding stage. The prior encoding of nontarget material may decrease attentional resources and increase memory load, thus leading to a less effective encoding of the final target list [50]. While Pastötter et al. evaluated this account by applying EEG during item encoding (see below), there is also behavioral support for the view. In particular, Weinstein et al. (2014) [51] used a multiple-list learning task in which participants were given a warning prior to the study of the final target list that there will be a later test on the target list. The warning did improve recall of the target list on that later test compared to when there was no such warning, suggesting that the prompt aided subjects in regaining attentional resources. In sum, the results provide evidence that retrieval processes can contribute to retroactive interference, and both encoding and retrieval processes can contribute to proactive interference (see also [48]).

### 2.5. Neural Correlates

Thus far, no previous imaging study has examined the neural processes underlying retroactive interference in multiple-list learning. However, three studies have applied EEG to examine the neural processes involved in proactive interference in multiple-list learning. The studies all found that prior encoding of nontarget material leads to an increase in oscillatory theta (5–8 Hz) and alpha (10–13 Hz) frequency bands [48,50,52]. Increases in theta activity in the human EEG have been argued to index memory load [53,54] and increases in alpha activity have been linked to inattention [55,56]. The findings thus indicate that memory load and inattention increase with the amount of prior encoding of additional study material, which may impair subsequent encoding of target material, and thus facilitate the buildup of proactive interference. Kliegl et al. (2015) [48] replicated the finding and additionally showed that individuals with high working-memory capacity reveal a less pronounced increase in theta power from nontarget to target lists than individuals with lower working-memory capacity, suggesting that individuals with high working memory capacity suffer less from interference-related memory load increases and inattention than individuals with lower working-memory capacity.

### 2.6. Interim Summary

In multiple-list learning, a failure at the retrieval stage has been argued to underlie both retroactive and proactive interference. In addition, a failure at the encoding stage may contribute to proactive interference. Regarding the retrieval stage, behavioral results from research analyzing response latencies as well as prior-list intrusions align with the view that additional nontarget encoding—be it before or after encoding of the target list—impairs memory search for the target items. Regarding the encoding stage, behavioral work indicates that decreasing attentional resources from the initial nontarget encoding to the later target encoding may also play a role in proactive interference. Imaging studies applying the proactive-interference variant of the task show oscillatory alpha and theta power—supposedly neural measures of inattention and memory load—increase with the amount of prior encoding of nontarget material, thus suggesting that the encoding of additional information impairs the later encoding of target information.

## 3. Mechanisms Underlying Inhibition-Induced Forgetting

### 3.1. Directed-Forgetting Tasks

**Figure 3:**
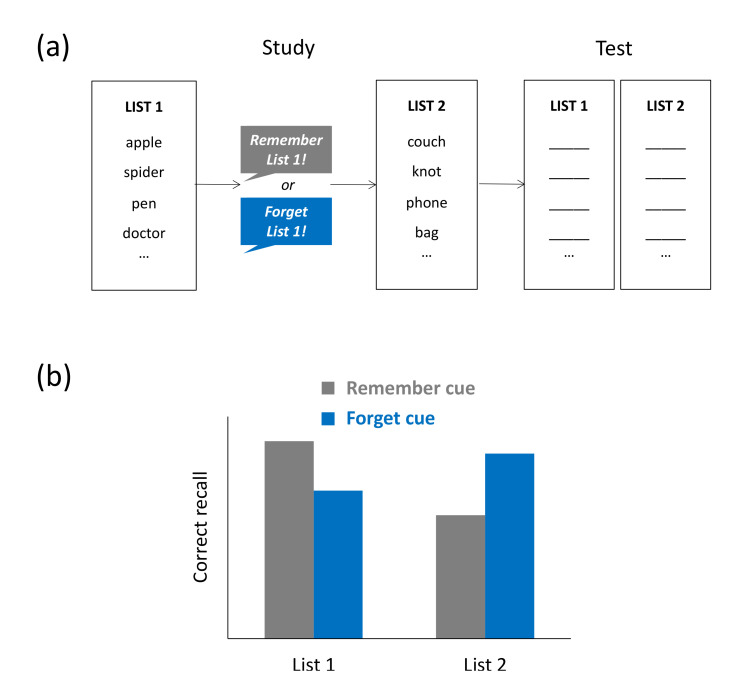
List-method directed forgetting task. (**a**) Typical task: Participants study two lists of items and, after study of list 1, they are asked to keep remembering the list for a later retention test or to forget the list altogether. After study of list 2—which is always to-be-remembered—subjects’ memory of both study lists is assessed, irrespective of original cuing. (**b**) Typical results: Compared to subjects who were cued to remember list 1, subjects who were were cued to forget list 1 show impaired recall of list 1 and enhanced recall of list 2.

While the findings on interference-induced forgetting discussed thus far show that the study of additional nontarget material can cause passive forgetting of target material, research on list-method directed forgetting (LMDF) suggests that interfering material can also be the `target’ of the forgetting itself, and inhibition is assumed to be the mechanism mediating this type of forgetting [20]. In a typical LMDF task, participants study two lists consisting, for instance, of unrelated nouns (e.g., *tree*, *iron*, *nose*). After studying list 1, they are either asked to keep remembering the just presented list (remember condition) or to forget the list because it will not be relevant for a later retention test (forget condition). List 2 is always to-be-remembered. During the test, participants’ memory for both lists is tested irrespective of original cuing. The results of that test typically show that recall of list-1 items is impaired and recall of list-2 items is improved in the forget condition, relative to the remember condition (see Figure 3a,b). In other words, providing an explicit instruction to forget seemingly irrelevant information can lead to impaired memory of that information and improved memory of subsequently studied information. LMDF has been observed across a wide variety of study materials, such as verbal [22], visual [57], and autobiographical material [58].

The view that LMDF reflects the action of an inhibitory mechanism that impairs access to the to-be-forgotten material is a key assumption of the so-called retrieval-inhibition account proposed by Bjork et al. [19,20,22]. The suggestion is that the forget cue triggers an inhibitory control process, which may operate during list-2 encoding, when the previously studied list-1 items may tend to become reinstated, thus causing proactive interference during list-2 encoding [59,60,61,62]. The assumption that the forget cue inhibits access to list-1 items can not only explain the observed reduction in list-1 recall performance but also accommodate for the observed enhancement in list-2 recall performance. In particular, if providing a forget cue reduces interference from list-1 items, recall of list-2 items during the final retention test should be facilitated and lead to higher recall in the forget than in the remember condition [47,63]. Because retrieval inhibition is assumed to reduce access to the whole list-1 study episode, the presence of list-1 forgetting should depend on the type of retention test used to assess memory. Free-recall tests, for example, provide no retrieval cues which might support reactivation of the list-1 study context, whereas reexposure of list-1 items during an item recognition test may support reactivation of the initial context. Behavioral findings are generally in line with this rationale, typically showing intact list-1 forgetting in free-recall tests and reduced or absent list-1 forgetting in item-recognition tests [22,64]. The findings on the role of testing format thus provide support for the retrieval-inhibition view.

**Figure 4:**
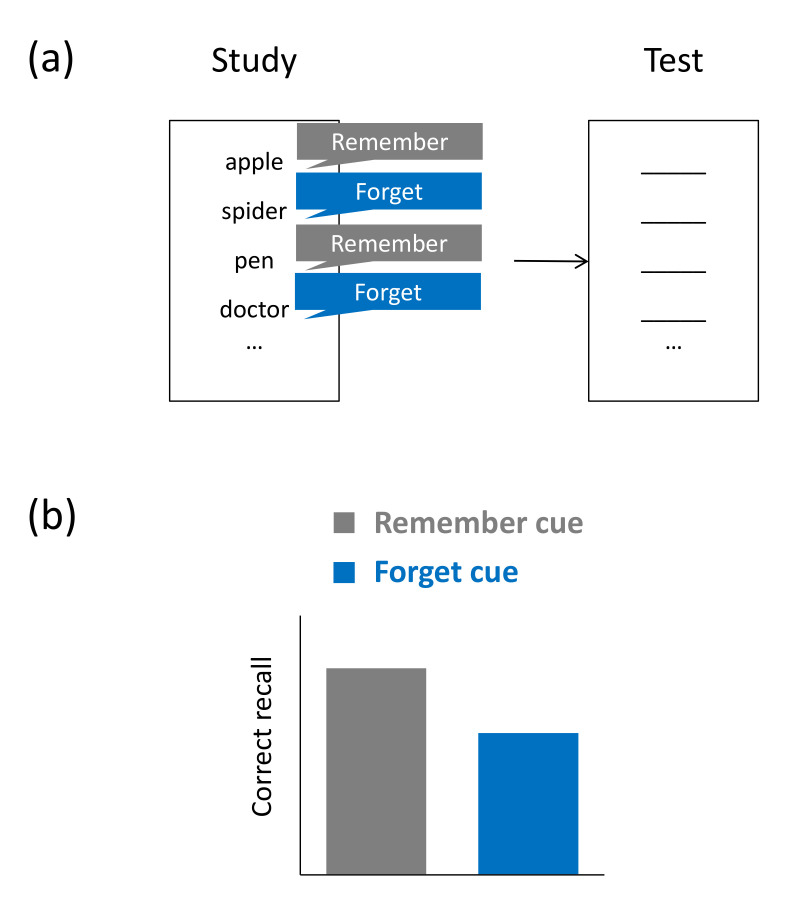
Item-method directed forgetting task. (**a**) Typical task: Participants study a list of items and, after studying each single item, they are either cued to remember or forget the item. On a subsequent retention test, subjects’ memory of all study items is assessed, irrespective of original cuing. (**b**) Typical results: Compared to subjects who were cued to remember list 1, subjects who were were cued to forget list 1 show impaired recall of list 1 and enhanced recall of list 2.

Findings from so-called item-method directed forgetting (IMDF) demonstrate that forget cues can also reduce intralist interference, i.e., interference effects arising from irrelevant material that is part of the same study list as the relevant material [65,66]. In a typical IMDF task, participants study only a single list of items (e.g., unrelated words) and, after presentation of each item, they are provided a cue to either forget or keep remembering the just presented word [67]. In a later retention test on all study items, participants typically recall fewer to-be-forgotten than to-be-remembered words (see Figure 4a,b). A prominent non-inhibitory account of IMDF assumes that to-be-forgotten words show lower recall rates because they experience less rehearsal than to-be-remembered words [68]. The idea is that an item is kept in working memory until either a forget or a remember cue is provided. A remember cue would induce effortful processing of the item, whereas a forget cue would halt the rehearsal process. If IMDF does reflect more elaborate encoding of to-be-remembered than to-be-forgotten items, then we would expect to find IMDF on a wide variety of test formats. Indeed, unlike LMDF, IMDF has been found to arise across many types of tests, including not only recall tests but also tests of item recognition and some implicit memory tasks [69].

However, more recently, the idea has gained traction that an additional, inhibitory mechanism—which has been referred to as encoding suppression [70]—may contribute to IMDF. According to this view, the forget cue can trigger active inhibitory mechanisms, which lead to a removal of forget-cued items from working memory [71,72]. This contrasts with the selective-rehearsal view stressing the role of processes acting on to-be-remembered material, which is assumed to be encoded more extensively than to-be-forgotten material. Behavioral findings showing that the forget condition requires more cognitive effort than the remember condition are, however, difficult to reconcile with the selective-rehearsal idea. For instance, forget instructions have been found to lead to slower reaction times on a concurrent secondary task [72,73] and stopping a motor response shortly after a forget cue has been provided is more successful than shortly after a remember cue has been provided [74]. The latter finding may indicate that the forget cue triggers analogous inhibitory mechanisms that work when a motor response is stopped. Imaging studies have provided further support for this notion (see next section).

### 3.2. Neural Correlates

Previous imaging work has examined LMDF using EEG and fMRI. These imaging methods were applied during item encoding. In addition, prior studies have applied repetitive Transcranial Magnetic Stimulation (rTMS), and transcranial Direct Current Stimulation (tDCS) during or before item encoding to gain further insights into the neural mechanisms underlying LMDF. Using EEG and analyzing brain oscillations, Bäuml et al. (2008) [59] found that the forget cue induces a decrease in phase coupling in the upper alpha frequency band (11–13 Hz), which was positively correlated with the amount of forgetting of list-1 items. Because alpha phase coupling between electrode sites has been argued to reflect the degree of synchrony between distant cell assemblies and coherent firing between distant cell assemblies is assumed to reflect a mechanism supporting binding processes in episodic memory [75], the observed decrease in phase coupling could reflect inhibitory unbinding of list-1 items and deactivation of the retrieval routes to list-1 items.

Hanslmayr et al. (2012) [76] investigated the neural mechanisms underlying LMDF by simultaneously recording participants’ EEG along with fMRI during item encoding. Similar to the Bauml et al. (2008) [59] study, results indicated a decrease in alpha phase coupling between electrode sites in the forget condition. The magnitude of the decrease was positively correlated with the amount of list-1 forgetting that was observed during the final test. In addition, fMRI analysis showed a BOLD signal increase in the forget condition, relative to the remember condition, in the dorsolateral PFC, a brain region repeatedly associated with memory control [77,78]. The BOLD signal increase showed a correlation with the decrease in neural synchrony in the forget condition and may thus represent another marker of the unbinding of forget-cued list-1 items via a prefrontally driven downregulation of the cortical network representing those items. Finally, the researchers demonstrated that direct rTMS stimulation of the dorsolateral PFC during encoding of list-2 items (i) increased the magnitude of list-1 forgetting during the final test and (ii) enhanced phase desynchronization, suggesting a causal link between neural activity in the dorsolateral PFC and list-1 forgetting.

More recently, Silas et al. (2016) [79] applied cathodal tDCS over the lateral PFC for 10 min immediately before subjects engaged in a typical LMDF task. While cathodal tDCS stimulation eliminated both list-1 forgetting and list-2 enhancement effects, a sham condition with no actual brain stimulation before completion of the LMDF task led to typical LMDF forgetting and enhancement effects. The researchers argued that tDCS stimulation of the lateral PFC led to a suppression of cortical excitability in this brain region, thereby interfering with the retrieval-inhibition mechanism. In sum, findings from neuroimaging and neurostimulation work on LMDF support the view that the forget cue triggers an inhibitory control process, which is mediated by the lateral PFC.

Regarding IMDF, several studies have used fMRI and even intracranial electrodes during item encoding to examine the neural mechanisms underlying this type of forgetting. The results from the fMRI work consistently indicate that during forget trials, there is increased activity in prefrontal brain regions [80,81,82,83,84]. Furthermore, hippocampal brain regions as well as surrounding parahippocampal areas have been found to show reduced activation in response to a forget cue. The results of connectivity analyses indicate that the reduction in hippocampal activity during forget trials may result from interactions with the PFC [81,82]. IMDF may thus be the result of a prefrontal mechanism, which downregulates encoding processes in the hippocampus.

Further support for this view comes from a recent study, in which 25 presurgical epilepsy patients with intracranial electrodes implanted in the dorsolateral PFC and the hippocampus completed an IMDF task [85]. Analysis of the EEG recordings showed that attempts to forget are associated with an increase in the lower theta (3–5 Hz) frequency band in the dorsolateral PFC and with a relatively wide frequency range in the theta, alpha, and beta (6–18 Hz) range in the hippocampus. Effective connectivity analyses showed an interaction between the dorsolateral PFC and the hippocampus in the beta frequency band (15–18 Hz) that was only reliable in the top-down direction. Critically, the control signal in the dorsolateral PFC was initiated 100–130 ms before it affected the hippocampus, thus providing evidence for a top-down inhibition that is both spatially and temporally specific.

## 4. Interim Summary

There is evidence that inhibitory mechanisms contribute to both LMDF and IMDF. In LMDF, the forget cue is assumed to impair access to the (to-be-forgotten) list-1 items without affecting the memory representation of those list items. Behavioral evidence for this retrieval-inhibition view comes from studies showing that LMDF typically arises in certain types of test formats—such as free recall—but not in others—such as item recognition. Results from imaging studies show that the forget cue induces a BOLD signal decrease in the dorsolateral PFC, which is correlated with a decrease in neural synchrony in the forget condition, possibly indexing the unbinding of forget-cued list-1 items. In addition, stimulation of the dorsolateral PFC via rTMS has been found to increase list-1 forgetting and cathodal stimulation of the lateral PFC via tDCS has been shown to largely eliminate list-1 forgetting, further underpinning a critical role of frontal brain regions for intentional forgetting. In IMDF, it has been suggested that the forget cue may induce active inhibitory processes that support the removal of forget-cued items from working memory. Behavioral studies showing, for instance, that forget cues lead to slower reaction times than remember cues on secondary tasks are consistent with this encoding-suppression view. Evidence from fMRI studies show that, in forget trials (i) prefrontal brain regions typically exhibit increased activity and (ii) hippocampal areas show reduced activity. A recent EEG study using intracranial electrodes provides evidence that, during forget trials, the dorsolateral PFC may induce top-down regulation of the hippocampus.

### 4.1. Retrieval-Practice Task

**Figure 5:**
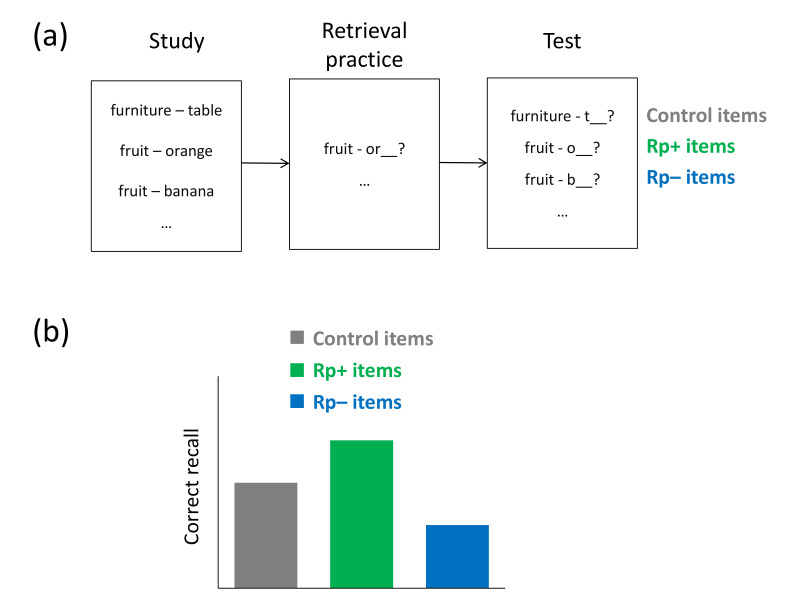
Retrieval-practice task. (**a**) Typical task: Participants first study items from different semantic categories before they are asked to retrieve repeatedly a subset of the exemplars from a subset of the categories. This retrieval practice creates three types of items: items from unpracticed categories (control items), practiced items from practiced categories (Rp+ items), and unpracticed items from practiced categories (Rp− items). On a final retention test, participants are asked to recall all items from the initial study phase. (**b**) Typical results: Recall of Rp+ items is often improved relative to control items, and recall of Rp− items is typically impaired relative to control items, reflecting retrieval-induced forgetting.

While prior research on directed forgetting suggests that interference from irrelevant material can be dealt with via an active cue to forget the material, research on retrieval-induced forgetting (RIF) indicates that inhibitory action can also be targeted at irrelevant material in a more automatic fashion. RIF is often examined using the retrieval-practice task, which was devised by Anderson et al. (1994) [23]. In this task, it is demonstrated that repeated retrieval of a subset of previously studied material can cause forgetting of related unpracticed material. In particular, subjects typically study items from different semantic categories (e.g., hobby-Running, hobby-Soccer, furniture-Table), before, in a subsequent retrieval-practice phase, they engage in a word-stem completion task in which a subset of the items from a subset of the categories has to be retrieved (e.g., fruit-Ru___). After a delay, typically filled with an unrelated distractor task, recall performance for all initially studied items is tested. The selective retrieval practice between study and test creates three types of items, usually labeled as Rp+ items, Rp− items, and control items. Rp+ items refer to practiced items from practiced categories (Running), Rp− items refer to unpracticed items from practiced categories (Soccer), and control items refer to items from unpracticed categories (Table). Recall of the Rp+ items is generally improved on the final test relative to the control items, and, more important, recall of Rp− items is typically impaired relative to the control items, thus reflecting RIF (see Figure 5a,b). Over the last 25 years, RIF has proven to be a very general effect and has been found to arise over a wide range of study material, including verbal [23], visuospatial [86], and autobiographical material [87], and also over a variety of experimental settings (for reviews, see [88,89]).

The inhibition account of RIF assumes that RIF arises because the memory representation of the Rp− items is directly affected [25]. This account proposes that during retrieval practice of some of the study items, related not-to-be-practiced items interfere and compete for conscious recall. To reduce this interference and to facilitate the selection of the to-be-practiced items, the memory representation of the not-to-be-practiced items becomes suppressed, which is why the inhibitory mechanism has also been referred to as item suppression [90]. For instance, when subjects are cued with hobby-Ru___ during the retrieval-practice phase, other hobbies such as Soccer may come to mind and thus compete for conscious recall. The account argues that, to reduce interference from Soccer, the memory representation of that item is suppressed and, as a result, recall of that item in the subsequent final test is impaired.

Because the inhibition account assumes that the suppression directly reduces the activation level of the competing memory representation itself, the occurrence of RIF should be largely independent of the type of test format. Evidence from prior research is generally in line with this implication, showing that RIF is not only observed with category-cued recall [23,91] but also arises in yes–no item recognition testing [92,93], and when receiver-operating-characteristic analysis is applied to investigate whether selective memory retrieval of Rp+ items impedes recognition of Rp− items [94,95] (for further evidence in support of the inhibition account, see [96]).

A related implication of the inhibition account is that RIF should arise irrespective of how Rp− items are cued on the final retention test. In line with this cue-independence assumption, prior research has indeed shown that RIF also arises when so-called independent probes are applied on the final retention test [91,96,97]. On such tests, an Rp− item (Soccer) is not tested with its initial cue from the study phase (hobby-S___) but with a completely novel cue instead (sports-S___). In the presence of such a novel retrieval cue, subjects may not draw on the original study cue to retrieve the Rp+ item, which should exclude potential blocking effects arising from Rp+ items on the final test as an explanation of RIF (for further elaboration on this argument, see [98,99]).

### 4.2. Neural Correlates

Several studies applying both EEG and fMRI have investigated the neural processes underlying RIF by examining neural correlates of the forgetting effect during the retrieval-practice phase. A prominent extension of the inhibition account emphasizes that item suppression may be the consequence of executive control processes, assuming that frontally mediated inhibitory control processes are applied to suppress competing information during retrieval practice, resulting in increased activity in these frontal areas. One early fMRI study provided critical evidence for the involvement of the prefrontal cortex in RIF by showing that repeated retrieval practice of Rp+ items leads to dynamic reductions in the anterior cingulate cortex (ACC) and the lateral PFC [100]. Because the ACC has been linked to interference detection and the lateral PFC has been linked to inhibition, the findings suggest that repeated retrieval practice may reduce demands on both interference detection and inhibition. In a subsequent fMRI study, Wimber et al. [101] essentially replicated the finding that both the ACC and the lateral PFC are responsive to retrieval practice. In addition, these researchers were able to show that activity in the ACC and the lateral PFC predicts the magnitude of the RIF effect. A relatively recent fMRI study found even more direct evidence that RIF can be attributed to an active inhibitory mechanism, revealing that retrieval practice of some items (in this case, images of either faces or objects) measurably reactivated competing (Rp−) items and then progressively suppressed those interfering competitors [102]). Importantly, the competitors’ cortical traces were suppressed even below the activity observed for control items, which supports the item-suppression idea.

Consistent with the findings from these fMRI studies, an EEG study found sustained event-related potentials over frontal brain areas in the retrieval-practice condition, relative to a restudy condition. In addition, the sustained event-related potentials were correlated with the amount of RIF that arose in the subsequent final test phase (Johansson et al., 2007 [103]). One further EEG study investigating oscillatory correlates of RIF demonstrated that theta oscillations (5–9 Hz) contribute to the effect. The origin of these oscillations was in the ACC, presumably mirroring the modulation of interference during retrieval practice [104]. In another study, Rp+ and Rp− items were stored in separate cerebral hemispheres, and selective retrieval of a given Rp+ item was associated with increased alpha/beta power (11.5–20 Hz) over the hemisphere where the sensory representation of the Rp− item was located [105]. Because increases in oscillatory alpha/beta power are generally considered to reflect cortical inhibition [106], the findings support the view that active inhibitory mechanisms are engaged during retrieval practice of Rp+ items. Overall, the findings from both fMRI and EEG studies are consistent with the view that the critical mechanism that causes RIF acts during the retrieval-practice phase, and not during the final-test phase, which is well in line with the inhibition account.

Applying EEG and fMRI, several studies have also examined how retrieval practice affects neural processes during the final memory test [107,108]. On the basis of the inhibition account, the prediction arises that neural markers should exist reflecting a reduced memory strength of Rp− items relative to the control items. In particular, the account assumes that during retrieval practice of the Rp+ items, the Rp− items interfere and, as a consequence, the inhibitory mechanism acts to reduce the Rp− items’ absolute strength in memory. An fMRI study by [108] showed that retrieval of Rp− items was associated with increased activation of the ventrolateral PFC, which is consistent with the inhibition account because increased activity in this region has been argued to reflect the retrieval of weakly bound memory traces [109]. Spitzer et al. (2008) [107] examined electrophysiological correlates of RIF on recognition memory and found that recall of Rp− items was associated with reduced early evoked theta power (4–7 Hz) and reduced gamma power (60–90 Hz). These findings are also well in line with the inhibition account because both of these effects have been suggested to reflect reduced memory signals for Rp− items.

### 4.3. Interim Summary

The inhibition account of RIF assumes that not-to-be-retrieved items interfere during selective memory retrieval of previously studied material and are inhibited to reduce the interference. This type of inhibition has been argued to affect the memory representation of the irrelevant material itself. Behavioral research has provided evidence for such item suppression by showing, for instance, that RIF arises across a wide variety of final retention tests. Previous results from studies applying EEG and fMRI show that, in line with the inhibition account, the critical mechanism underlying RIF occurs during the retrieval-practice phase and involves the dorsolateral PFC and the ACC. Additional EEG and fMRI work also supports the item-suppression view by showing reduced evoked theta and gamma power associated with the competing items at the time of the test and increased activation in the ventrolateral PFC.

## 5. Conclusions

Forgetting of relevant material can occur because passive environmental conditions change. The most widely examined passive cause of forgetting is interference-induced forgetting (but see recent research on decay as another potential cause of passive forgetting; in particular [110,111]) and the two most commonly applied tasks to examine interference-induced forgetting are paired-associate learning and multiple-list learning. Cognitive studies suggest that similar mechanisms can underlie the interference-induced forgetting of relevant material in the two experimental tasks. In particular, there is evidence from both paired-associate learning and multiple-list learning suggesting that memory of the relevant material can be impaired during testing because additional, irrelevant study material is coactivated, leading to a less focused and less effective memory search for the relevant material. For paired-associate learning, recursive reminding during encoding appears to constitute a factor that can modulate the magnitude of the interference effect. For the proactive-interference version of multiple-list learning, impaired attentional encoding of the relevant material resulting from prior study of irrelevant material can play an additional role for interference-induced forgetting.

Results from PET and fMRI research suggest that interference effects observed in paired-associate learning involve the lateral PFC, a brain region that has been argued to play a critical role when conflict between competing responses needs to be resolved. Additional fMRI work has been found, suggesting that hippocampal brain regions and the medial PFC may be involved in remembering and memory integration, and may thus be critical for the modulation of interference effects. EEG studies using the proactive-interference version of the multiple-list learning task have shown that oscillatory alpha and theta power, which may reflect the degree of inattention and memory load, increase with the amount of previously studied irrelevant material, thus providing evidence that impaired encoding contributes to (proactive) interference effects in this task.

The results from behavioral and imaging work demonstrate some consistency regarding the cognitive and neural mechanisms underlying interference effects in paired-associate and multiple-list learning. In paired-associate learning, behavioral studies suggest that the additional learning of irrelevant material may block retrieval of the relevant material during tests and fMRI findings suggest that activity in the lateral PFC increases with the number of irrelevant responses during tests, potentially reflecting issues at selecting the relevant material in the presence of irrelevant material. In multiple-list learning, behavioral studies showing that the preceding encoding of irrelevant material impedes effective encoding of the subsequently studied relevant material, and EEG work demonstrating that the preceding encoding of irrelevant material affects measures, which are assumed to reflect impaired encoding support the view.

While the findings on the cognitive and neural mechanisms underlying interference effects in the two tasks thus show a relatively compatible pattern, there are still a number of empirical gaps that need to be addressed in future work. For instance, for paired-associate learning, behavioral studies thus far have mostly focused on the role of retrieval processes for interference effects, but very few behavioral studies have examined how encoding mechanisms may contribute to interference in this task. In contrast, imaging work applying paired-associate learning has been more focused on the encoding factors underlying interference effects, but there has been little research on potential neural markers of retrieval processes. For multiple-list learning, the number of both behavioral and neural studies is relatively limited thus far but a similar pattern arises as with paired-associate learning, i.e., behavioral studies have been mostly concerned with retrieval mechanisms and imaging studies have been focused on encoding mechanisms. Thus, for both tasks, more behavioral research is still required to investigate the cognitive mechanisms underlying interference during item encoding, and more imaging research is needed to fully understand the neural mechanisms underlying interference during retrieval.

Forgetting can also occur because active inhibitory mechanisms target previously studied irrelevant material. Three prominent tasks with which such inhibitory mechanisms have been examined are the LMDF task, the IMDF task, and the retrieval-practice task. Regarding the LMDF task, there is behavioral evidence that the irrelevant list-1 material is inhibited when a cue to forget the list is provided. However, because the forgetting is typically limited to certain types of tests, such as free recall and cued recall, but does not arise in item recognition, it has been argued that the forget cue does not affect the memory representation of the irrelevant material itself, but rather impairs access to the list’s retrieval route. Inhibitory processes have also been suggested to contribute to the forgetting observed in the IMDF task because behavioral studies have shown that to-be-forgotten material requires more cognitive effort than to-be-remembered material. For the retrieval-practice task, there is ample evidence from behavioral studies showing that the forgetting arises across a wide variety of final retention tests, including item recognition and independent-probe tests, which suggests that retrieval practice can trigger inhibitory processes that affect the memory representation of the irrelevant, unpracticed material in memory itself.

Neurally, findings from EEG and fMRI work point to a critical role of the dorsolateral PFC for LMDF, as forget cues have been found to induce a BOLD signal increase in this brain region, which is correlated with a decrease in neural synchrony, a pattern that may indicate inhibition of the list-1-unit. Additional results from rTMS and tDCS further support the idea that the dorsolateral PFC is involved in this type of forgetting. FMRI studies also point to a critical role of the lateral PFC for IMDF, with fMRI studies suggesting that forget cues increase lateral PFC activity, which may then downregulate hippocampal activity. Finally, considerable knowledge from EEG and fMRI work has accumulated on the neural correlates of RIF. These studies align with the item-suppression view, as they suggest that the mechanism underlying RIF acts during the retrieval-practice phase and involves the dorsolateral PFC. EEG and fMRI studies examining neural markers of forgetting on the later retention test also support the item-suppression view by showing that, during retrieval of irrelevant material, neural markers are observed, which have been argued to reflect weakly bound memories.

All in all, behavioral and imaging studies show a high level of consistency regarding the cognitive and neural mechanisms underlying forgetting in the three single experimental tasks. For LMDF, behavioral work suggests that retrieval inhibition acts during list-2 encoding and imaging work supports the view by showing that potential neural markers of inhibition, such as increased activity in the dorsolateral PFC and a decrease in neural synchrony, can be observed during list-2 encoding. For IMDF, behavioral studies indicate that an active inihbitory process may at least play an additional role for the forgetting, and imaging studies align with this idea, suggesting that the lateral PFC may downregulate hippocampal activity in response to a forget cue. Finally, for RIF, there is solid evidence from behavioral work indicating that during selective memory retrieval, irrelevant material becomes inhibited, with imaging work supporting this view, suggesting that during retrieval practice, the lateral PFC acts to induce a reduced activation level of the irrelevant material’s memory representation.

Research examining inhibitory processes by applying the LMDF, IMDF, or RIF paradigms converges on the view that the lateral PFC is critically involved in inhibitory action. Future work still needs to investigate how exactly the lateral PFC induces inhibition in each of the three experimental tasks. In particular, additional research should focus on how the pathways through which a forget cue induces either LMDF or IMDF differ and in what sense they are similar. For instance, while previous work suggests that forget cues can lead to a downregulation of hippocampal areas via the lateral PFC, it remains largely unclear if and how hippocampal regions are involved in LMDF. Furthermore, on a much more general level, further work is needed to determine the exact role of the hippocampus for long-term memory. Indeed, while the classic view holds that the hippocampus is critical for the consolidation of memories [112], a more recent perspective rather emphasizes that the area may be involved in binding item information to context information [113]. Greater clarity regarding the role of the hippocampus would certainly have broad implications for theorizing about the area’s possible significance for active (and passive) forgetting. Furthermore, while in RIF, the ACC has been argued to detect interference during selective memory retrieval and the lateral PFC has been suggested to resolve the interference via inhibition, further research is still required to investigate the exact pathways through which selective retrieval mediates inhibition. Finally, while research on RIF has examined neural markers of the forgetting on the final test, no such research has yet been conducted in LMDF and IMDF.

In summary, memory research has accumulated considerable behavioral and neural evidence, suggesting that forgetting can result from both passive and active causes. While interference as a passive cause of forgetting may be undesirable in many situations, there are active tools that can be applied to prevent interference effects, such as implementing retrieval practice of the previously studied material during the study of several lists or units (for a review, see [114]). In this regard, optimizing our memory to deal with the information overload that we encounter in our daily lives involves methods that concern the active (and potentially intentional) suppression of irrelevant material as well as methods that can alleviate the detrimental effects of passive forgetting of relevant material.
